# A role for E-cadherin in ensuring cohesive migration of a heterogeneous population of non-epithelial cells

**DOI:** 10.1038/ncomms8998

**Published:** 2015-08-14

**Authors:** Kyra Campbell, Jordi Casanova

**Affiliations:** 1 Institut de Biologia Molecular de Barcelona (CSIC), C/Baldiri Reixac 10, Barcelona, Catalonia 08028, Spain; 2 Institut de Recerca Biomèdica de Barcelona C/Baldiri Reixac 10, Barcelona, Catalonia 08028, Spain

## Abstract

Collective cell migration is a key process underlying the morphogenesis of many organs as well as tumour invasion, which very often involves heterogeneous cell populations. Here we investigated how such populations can migrate cohesively in the *Drosophila* posterior midgut, comprised of epithelial and mesenchymal cells and show a novel role for the epithelial adhesion molecule E-cadherin (E-Cad) in mesenchymal cells. Despite a lack of junctions at the ultrastructure level, reducing E-Cad levels causes mesenchymal cells to detach from one another and from neighbouring epithelial cells; as a result, coordination between the two populations is lost. Moreover, Bazooka and recycling mechanisms are also required for E-Cad accumulation in mesenchymal cells. These results indicate an active role for E-Cad in mediating cohesive and ordered migration of non-epithelial cells, and discount the notion of E-Cad as just an epithelial feature that has to be switched off to enable migration of mesenchymal cells.

## Abstract (web summary)

Collective cell migration underlies the morphogenesis of many organs, and often involves heterogeneous cell types. Here the authors show a role for the adhesion molecule E-cadherin in maintaining the attachment of mesenchymal cells during *Drosophila* posterior midgut development.

## Graphical abstract

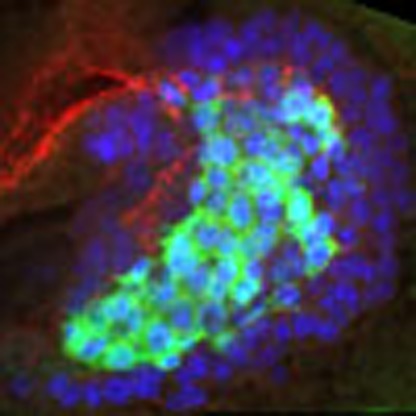

The fundamental role of cell migration in development and homeostasis has been recognized for quite some time now. In particular, the fact that static cells acquire migratory capacity and become motile at very precise times and settings and, conversely, that inappropriate migration is associated with many pathologies. Very often, both in normal and disease conditions, collective migration involves heterogeneous cell populations with distinct mesenchymal and epithelial features. However, it is poorly understood how such populations can migrate cohesively.

We have addressed this issue by analysing endoderm migration in the process of gut formation. The endoderm of *Drosophila* is subdivided into three populations of cells before migration that differ morphologically and genetically (Fig. 1a; refs 1, 2). Throughout migration, principle midgut epithelial cells (PMECs) are apicobasally polarized, columnar and regular in shape, although without adherens junctions (Fig. 1a,b; refs 2, 3). Previous work has shown that these cells are in direct contact with the neighbouring mesoderm, and require the mesoderm as a substrate for migration^2^. In contrast, interstitial cell precursors (ICPs) and adult midgut precursors (AMPs) have been shown to be mesenchymal throughout migration, as seen by their irregular morphology, lack of apicobasal polarity and extensive protrusive activity (Fig. 1b,c; refs 2, 3, 4), and this is particularly apparent in live movies where they are seen to extend and retract many protrusions, constantly making and breaking contacts with each other and the surrounding PMECs (Fig. 1c; Supplementary Movie 1). Migration of midgut cells is highly coordinated; indeed, ICP and AMPs require PMECs for migration, as when PMECs are genetically ablated, ICPs and AMPs completely fail to migrate^1^. However, similar experiments showed that migration of PMECs does not rely on interactions with the ICPs^1^. While it is known that the coordination of PMEC behaviour with the mesoderm is mediated by integrins^5^,^6^, how ICP and AMP behaviour is coordinated with PMECs, is completely unknown.

We previously identified a set of specific GATA factors in *Drosophila* and mammals that are responsible for inducing epithelial cells towards a migratory endoderm behaviour^7^ and, interestingly, these mesenchymal cells keep low levels of E-cadherin (E-Cad) protein throughout migration. While it is widely recognized the fundamental impact of E-Cad on cell behaviour, there is an important debate about its functional role. Considered for a long time to be a protein that assured the static behaviour of epithelial cells, with the repression of E-Cad long considered a necessary or even a sufficient step for epithelial cells to become migratory through an epithelial-to-mesenchymal transition (EMT). Indeed, the switch from E-Cad to an alternative cadherin protein has been claimed to be a critical event in such processes^8^, with cadherins such as N-Cad playing active roles in mesenchymal cell migration^9^. Thus, it is intriguing that mesenchymal–endodermal cells express E-Cad, but is unclear whether this is simply a remnant of an incomplete EMT or whether E-Cad may actually play an active role in the migrating endoderm.

Thus, we decided to investigate the putative role of E-Cad in the heterogeneous population of endodermal cells in the *Drosophila* midgut, where E-Cad is expressed not only in the polarized PMECs but also in the non-polarized ICP and AMPs throughout migration (Fig. 1b; Supplementary Fig. 1). Our results show a functional requirement for E-Cad for the cohesive migration of mesenchymal cells. Furthermore, we have also found that the recycling mechanisms and polarity proteins thought to be specific to polarized epithelial cells can be shared by non-epithelial and non-apicobasally polarized cells. Thus, in this regard, our data challenge the paradigm of E-Cad as just an epithelial feature that has to be switched off to enable the migration of mesenchymal cells.

## Results

### E-Cad is required for the collective migration of midgut cells

To examine a possible role for E-Cad in the behaviour of the mesenchymal cells during midgut migration, we focused our studies on the larger ICPs, which are clearly distinguishable by their big nuclei, and because they take up stereotypic positions during migration. It is not possible to analyse gut migration in the complete absence of E-Cad, due to the requirement for its maternal contribution during oogenesis and very early embryonic development, and because its cell-specific downregulation by RNA interference does not work at this early embryonic stage. Thus, we chose to focus our analysis on the strong allele *shg*^*G317*^, which shows a phenotype stronger than the zygotic null mutant alleles, and an extensive genetic characterization suggests this is due to a dominant-negative effect on the maternal contribution^10^. This was reinforced by staining for E-Cad in *shg*^*G317*^ embryos, which showed that E-Cad expression is reduced to almost undetectable levels in the migrating posterior midgut (PMG) (Supplementary Fig. 2a). Furthermore, this allele was previously used to uncover a role for E-Cad in the repolarization of the endoderm after migration, and shown to give similar, but more defined phenotypes as seen in the genetic null allele *shg*^*IH*^ (ref. 2).

**Figure 1:**
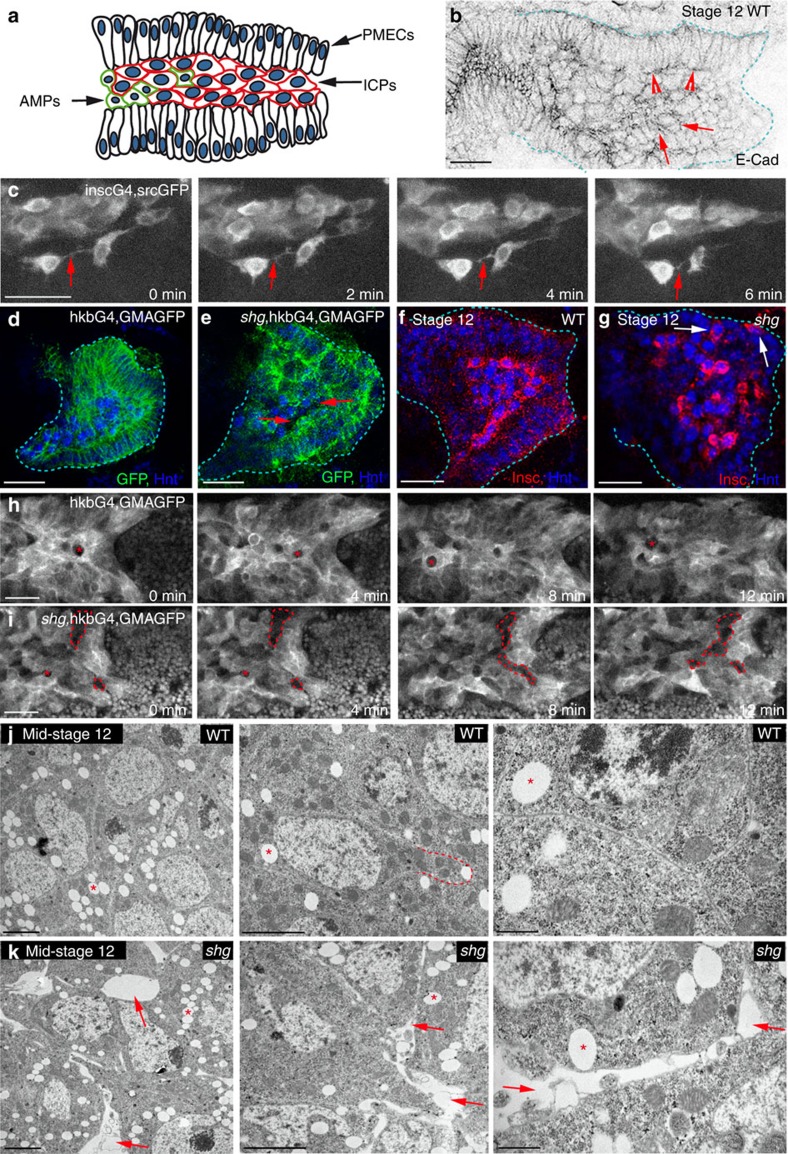
E-Cad is required for the highly cohesive behaviour of PMG cells during migration. (**a**) The migrating PMG consists of three groups of cells: PMECs, ICPs and AMPs. (**b**) Wild-type (WT) embryo stained for E-Cad, arrowheads point to PMECs, arrows to ICPs. The PMG is demarcated by dashed lines. (**c**) Stills from 20-min movies of ICPs visualized by a membrane-bound GFP driven by insc-Gal4, arrows point to protrusions. (**d**–**j**) Stage 12 WT (**d**,**f**,**h**,**j**) and *shotgun (shg)* (**e**,**g**,**i**,**k**) mutant embryos (E-Cad is encoded by the gene *shg*). The PMG is demarcated by dashed lines in **d**,**e**,**f**,**g**. (**d**,**e**,**h**,**i**) GMAGFP is used to label cortical actin^11^, in both fixed (**d**,**e**) and *in vivo* movies (**h**,**i**), arrows point to holes between cells in *shg* mutants (**e**); in *shg* mutants, PMECs show some defects in apicobasal polarity (**e** and ref. 2). (**h**,**i**) Asterisks mark the positions of unmarked germ cells, while gaps between cells appear as larger irregular holes (**i**, dashed lines). (**f**,**g**) PMG cells labelled for Hnt (blue), which is expressed in both PMECs and ICPs, and Inscuteable (Insc) (red), which is expressed in just ICPs, arrows point to mispositioned ICPs (**g**). (**j**,**k**) Transmission electron microscopy images of ICPs show no visible junctions between cells, arrows point to holes in *shg* mutants, asterisks to lipid droplets. Dashed line in **j** outlines a cell protrusion tightly aligned with neighbouring cells. Middle and right images are increasing magnifications from the same embryo. Scale bars, 20 μm (**b**–**i**); 2 μm (**j**,**k**, left and middle); 500 nm (**j**–**k**, right).

In wild-type embryos, midgut cells are highly organized throughout migration with PMECs forming two pseudostratified layers that adhere to the mesoderm on their basal sides, and sandwich the ICPs apically, which are tightly packed together (Fig. 1d,f). In E-Cad mutants, this stereotypical organization of midgut cells is lost with gaps appearing between the ICPs (Fig. 1e). Furthermore, ICPs are found both intermingled with and on the basal side of the PMECs (Fig. 1g), indicating perturbed migration. To further investigate cell dynamics, we labelled midgut cells with a marker for actin^11^ and followed their behaviour *in vivo* using two-photon microscopy. We found that in E-Cad mutants, the gaps and lack of cohesion both between the ICPs and between ICPs and PMECs increase and are more frequent as migration proceeds (Fig. 1h,i; Supplementary Movies 2 and 3), suggesting a continuous role for E-Cad in mediating attachment between migrating midgut cells.

### E-Cad is required for cohesion of the migrating midgut cells

The E-Cad requirement for the precise alignment of midgut cells throughout migration suggested the presence of intercellular junctions. However, ultrastructural analysis showed only a few scattered spot adherens junctions (sAJs) in a very small number of cells (Supplementary Fig. 3a), with the clear majority of midgut cells possessing no visible junctions (Fig. 1j), as was reported in a previous electron microscope study of cellular junctions in the early embryo^3^. Despite this lack of junctions, all midgut cell membranes in the wild type are extremely closely aligned, with ICPs protrusions tightly associated with the contours of neighbouring cells (Fig. 1j). Conversely, in E-Cad mutants, the cohesion between midgut cells is markedly altered; cell membranes are no longer aligned and large gaps appear between ICP cells (Fig. 1k), despite the fact that occasional sAJs can still be found (Supplementary Fig. 3b). Its worth noting that the lack of cohesion between ICPs occurs with no detectable change in the cohesion between the PMECs. Together, these results show that E-Cad is required for the proper adhesion of migrating midgut cells in the absence of distinct morphological junctions. This is in contrast with the adhesive function of E-Cad in epithelial cells, where it is most often associated with zonula or sAJs^12^,^13^.

### E-Cad is needed to coordinate migration of ICPs with PMECs

**Figure 2:**
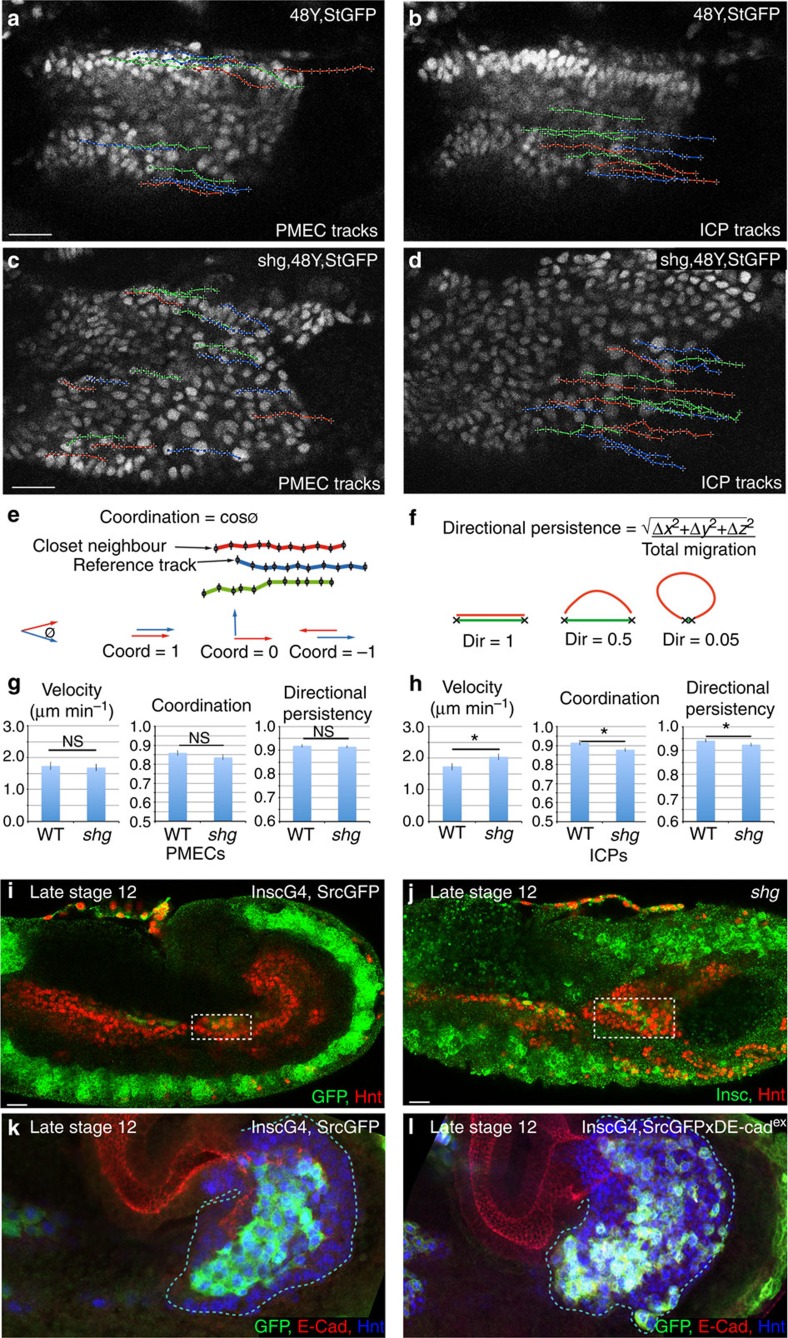
E-Cad is required to coordinate velocity and direction of migration between PMG cells. (**a**–**d**) Tracks representative of the paths taken by PMECs (**a**,**c**) and ICPs (**b**,**d**) in wild-type (WT) (**a**,**b**) and *shg* (**c**,**d**) mutant embryos, StingerGFP (StGFP) labels cell nuclei. PMECs and ICPs are identified by their nuclear diameter (PMECs <3.5 μm and ICPs >5.5 μm). To aid comparison, tracks are arbitrarily labelled red, blue and green. (**e**) Cell coordination is a correlation of the direction and magnitude of neighbouring tracks (defined by track start) at each time point. (**f**) Directional persistence is the shortest distance between start and end point, divided by the total migration track length. (**g**,**h**) Velocity, coordination and directional persistence values calculated from movies of WT and *shg* mutant embryos. Data are presented as mean ±s.e.m. *P<0.05; **P<0.01; NS, not significant; by paired *t*-test, *n*=6 for each condition (see Supplementary Table 1 for raw data). (**i**,**j**) ICPs position (outlined by dashed box) in late stage 12 embryos visualized in WT by insc-Gal4, SrcGFP (insc is specific to the ICPs and AMPs in the midgut but also drives in some other tissues, see Methods) (**i**) or antibody staining for insc (**j**). ICPs are mispositioned in *shg* mutants. (**k**,**l**) Late stage 12 embryos, with insc-Gal4 driving expression of either srcGFP alone (**k**) or together with the E-Cad dominant-negative construct dE-cad^ex^ (**l**). Expression DE-cad^ex^ causes the stereotypic positioning of migrating ICP cells to be lost (compare **l** with WT **k**), arrows point to mispositioned ICP cells. Scale bar, 20 μm. coord, coordination; dir, directional persistence.

To further investigate the role of E-Cad in the migration of midgut cells, we tracked the nuclei of subsets of PMEC and ICP cells using a combination of custom-built ImageJ macros that allowed the automated three-dimensional (3D) tracking and manual validation of each track (see Methods). These analyses revealed a remarkable coordination of PMG cell migration as the ratio of velocity between ICPs and PMECs within an embryo is always close to 1 (Supplementary Fig. 4). In wild type, both PMECs and ICPs migrate very directionally, with a high degree of coordination and a velocity of 1.7 μm min^−1^ (Fig. 2a,b,e–h; Supplementary Movie 4; see Supplementary Table 1 for raw data). In E-Cad mutant embryos, the migration speed, coordination and directional persistence of PMECs are unaffected (Fig. 2c,g; Supplementary Movie 5). In contrast, ICP migration is significantly altered: they migrate faster than in wild type; they show a lower degree of coordination both with each other and with the PMECs; and they no longer migrate along a straight path (Fig. 2d,h; Supplementary Movie 5). Thus, the ratio of velocity between ICP and PMECs is perturbed (Supplementary Fig. 4), and consequently the ICPs end up mispositioned (Fig. 2i,j). This result suggests that ICPs have a higher intrinsic migration capability than PMECs, and that E-Cad-mediated adhesion between ICPs and PMECs lowers ICP speed and helps coordinate their joint migration.

To ensure that the effects on midgut migration are due to a specific requirement for E-Cad within PMG cells, we expressed a dominant-negative E-Cad construct (DE-cad^ex^ (ref. 14)), using the driver 48Y-Gal4, which targets it specifically to the PMG cells. This construct has previously been extensively characterized and shown *in vivo* to act as a strong dominant negative when expressed in the background of endogenous E-Cad, without any ‘side effects' on Wingless signalling^14^. Similar to E-Cad mutants, we find that this produces gaps between ICP cells, indicating a loss of adhesion (Supplementary Fig. 2b). Furthermore, live analyses revealed that while the expression of a dominant-negative E-Cad does not affect the migratory behaviour of PMECs (Supplementary Fig. 2c,e; Supplementary Movie 6), ICPs migrate significantly faster than in wild type (Supplementary Fig. 2d,f), indicating that as in *shg* mutants ICPs and PMECs lose their coordination of migration velocity.

Finally, to ensure that changes in ICP migration are due to a specific requirement in these cells, and not just due to subtle undetected changes in the PMECs, we expressed dominant-negative E-Cad using the driver insc-Gal4, which targets it specifically to the ICPs (Fig. 2k). Similar to E-Cad mutants, the stereotypical organization of the ICPs is lost, and they are found both intermingled with and on the basal side of the PMECs (Fig. 2l, compare with E-Cad mutant in Fig. 1g). Thus, these results support a role for E-Cad specifically in the mesenchymal ICP cells, to coordinate their migration with both themselves and the surrounding PMECs.

### High levels of E-Cad slows migration of both ICPs and PMECs

**Figure 3:**
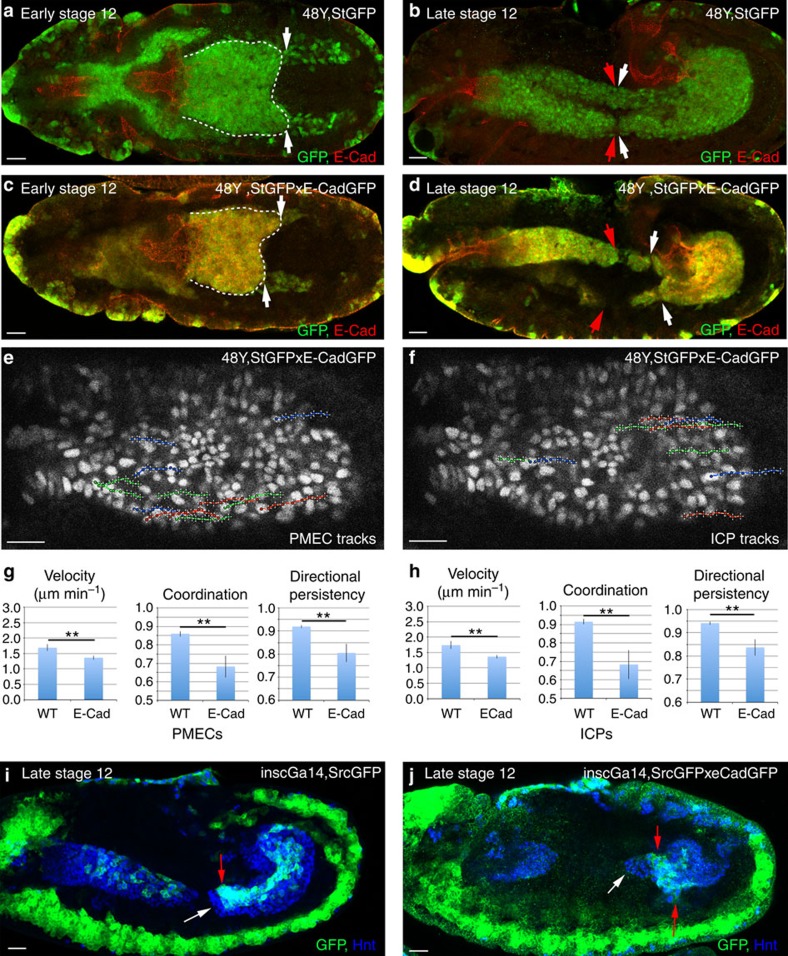
Overexpression of E-Cad delays the migration of both PMECs and ICPs. (**a**–**d**) Early stage 12 (**a**,**c**) and late stage 12 (**b**,**d**) embryos, with 48Y-Gal4 driving expression of either StGFP alone (**a**,**b**) or together with E-Cad-GFP (**c**,**d**). (**a**,**c**) Dashed lines delineate the PMG, whose bilateral symmetry is lost in **c**. (**b**,**d**) In wild-type (WT) embryos, the anterior midgut (red arrows) and PMG (white arrows) have met (**b**), while this is delayed by increased E-Cad levels (**d**). (**e**,**f**) Representative tracks of the paths taken by PMECs (**e**) and ICPs (**f**) with increased levels of E-Cad. (**g**,**h**) Velocity, coordination and directional persistence values calculated from movies of WT and E-Cad overexpressing PMGs. Data are presented as mean ±s.e.m. *P<0.05; **P<0.01; by paired *t*-test, *n*=6 for each condition (see Supplementary Table 1 for raw data). (**i**,**j**) Late stage 12 embryos, with the ICP-specific Gal4, inscG4, driving expression of either srcGFP alone (**i**) or srcGFP and E-Cad-GFP (**j**). White arrow indicates the migration front of PMECs, and red arrow of the ICPs. Scale bar, 20 μm.

On specification, midgut cells lower their levels of E-Cad as compared with the neighbouring epithelial cells^7^. To test the functional relevance of the levels of E-Cad, we overexpressed E-Cad throughout the midgut during migration (see Methods). Indeed, higher levels of E-Cad caused gross defects as the midgut loses its bilateral symmetry, and shows a significant delay in fusing with the anterior midgut (Fig. 3a-d). A detailed *in vivo* analysis showed that higher levels of E-Cad reduce the speed of migration in both PMECs and ICPs, without affecting their coupling (Fig. 3e–h; Supplementary Fig. 4; Supplementary Movie 7). Increased E-Cad also causes a decrease in the coordination and directional efficiency of both cells types, which reflects the change of the migration path from straight to more jiggly (Fig. 3e–h). To ensure that the effects on ICP migration are not just a consequence of changes in PMEC behaviour, we overexpressed E-Cad using the driver insc-Gal4 and found it sufficient to slow down ICPs, which no longer migrate in parallel with the PMECs, and are found lagging behind (Fig. 3j). This further supports the notion that ICPs undergo an active migration and that levels of E-Cad-mediated adhesion ensure a balance between coupling and efficient migration of the two cell types.

### E-Cad undergoes endocytic trafficking in PMG cells

**Figure 4:**
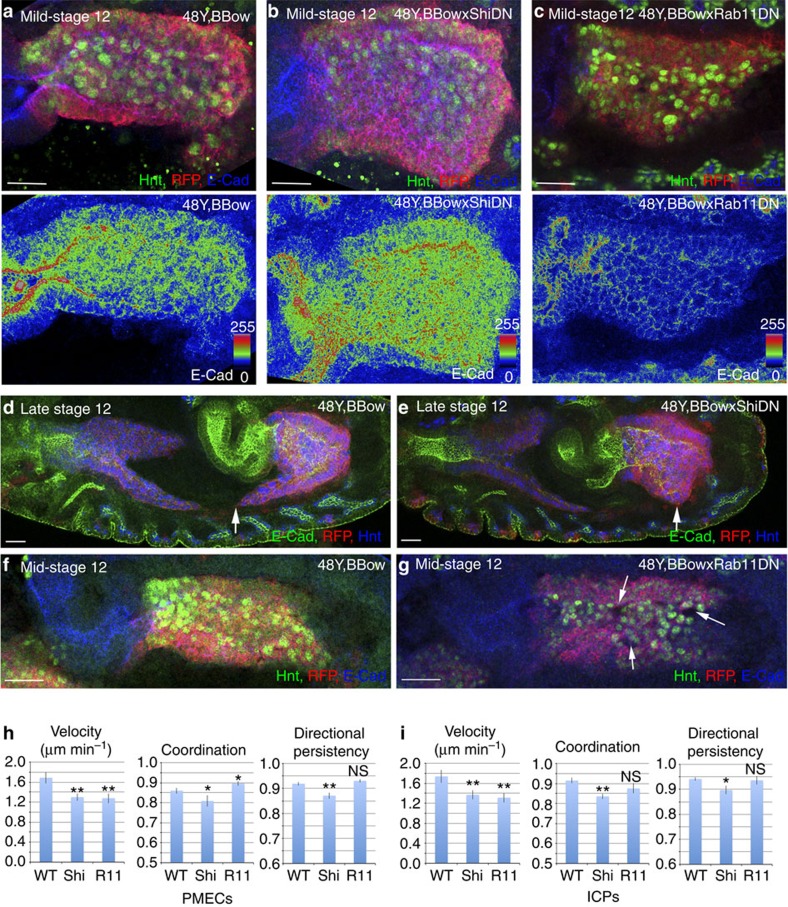
E-Cad is endocytosed and recycled in both PMECs and ICPs throughout migration. (**a**–**g**) Genotypes are 48Y,UAS-Bbow (**a**,**d**,**f**); 48Y,UAS-Bbow;UAS-Shi^DN^ (**b,e**) and 48Y,UAS-Bbow;UAS-Rab11^DN^ (**c**,**g**). (**a**–**c**) PMG cells are visualized by staining for Hnt (green) and their membranes are marked by the BBow construct (red fluorescent protein (RFP), red). (**a**–**c**) A colorimetric readout of E-Cad levels. (**d**,**e**) Ectopic Shi^DN^ delays the migration of the PMG. Arrows point the migration front. (**f**,**g**) Blocking Rab11 activity causes holes at the ICP–ICP and ICP–PMEC boundaries (**g**, arrows). (**h**,**i**) Velocity, coordination and directional persistence values calculated from movies made of WT, Shi^DN^ and Rab11^DN^ expressing PMGs. Data are presented as mean ±s.e.m. *P<0.05; **P<0.01; NS, not significant; by paired *t*-test, *n*=6 for each condition (see Supplementary Table 1 for raw data). Scale bar, 20 μm.

In epithelial cells, E-Cad is dynamically endocytosed and recycled to modulate cell–cell contacts while maintaining overall adhesion^15^,^16^,^17^. We reasoned that a similar mechanism might also operate in the non-epithelial midgut cells, as E-Cad is very dynamic in both PMECs and ICPs, forming punctate spots on the membranes that are constantly appearing and disappearing^7^. To test this, we used a dominant-negative form of dynamin (Shi^DN^), which is required for the scission of endocytic vesicles and recycling endosomes^18^,^19^,^20^. In fact, midgut expression of Shi^DN^ caused a marked upregulation of membrane E-Cad in both PMECs and ICPs (Fig. 4b). Conversely, the levels of E-Cad are greatly lowered throughout the midgut on expression of a dominant-negative form of Rab11, which impairs the recycling of endocytosed proteins^21^ (Fig. 4c). These results thus indicate that also in the non-epithelial midgut cells E-Cad is dynamically endocytosed and recycled.

**Figure 5:**
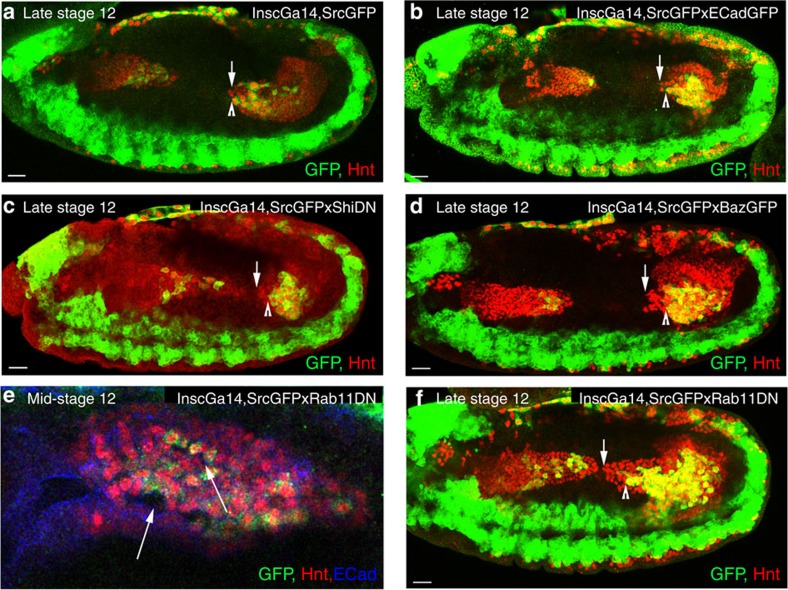
Increasing E-Cad levels or interfering with endocytosis specifically in the ICPs causes mutant phenotypes similar to the effects seen when triggered in all midgut cells. (**a**–**f**) ICP cells are visualized at 50% germband retraction (**a**–**d**,**f**) or in mid-stage 12 embryos (**e**) by staining for insc-Gal4, SrcGFP (GPF) and Hnt (red), while PMECs express Hnt (red) alone. (**a**) in wild-type (WT) embryos, the ICPs (arrow head) and PMECs (arrow) have migrated in parallel to the middle of the embryo. (**b**–**d**) Increasing E-Cad levels specifically in the ICPs through expression of E-Cad-GFP (**b**), ShiDN (**c**) or Baz-GFP (**d**) delays the migration of both ICPs and PMECs, which no longer migrate in parallel. (**e**) Blocking endocytic recycling through the expression of Rab11DN specifically in the ICPs causes holes to appear both between the ICPs (arrow), and between ICP and PMECs (arrow head). (**f**) Expression of Rab11DN specifically in ICPs slows the migration of ICPs (arrow head) without affecting the migration of the PMECs (arrow). Scale bar, 20 μm.

While blocking endocytosis or recycling is likely to alter more than E-Cad trafficking, we also analysed the functional consequences of blocking endocytosis. We found a significant delay in PMG migration on expression of the Shi^DN^ construct (Fig. 4e). Indeed, live-cell tracking revealed that blocking endocytosis causes a decrease in velocity of both cell types, and a loss of coordination and directional persistence, thus having a milder yet similar effect to overexpression of E-Cad (Fig. 4h,i; Supplementary Fig. 5a,b; Supplementary Movie 8). It is interesting to note that either overexpression of E-Cad or Shi^DN^ causes a delay in not only ICP migration but also in the PMECs (Fig. 5b,c). Conversely, blocking recycling with the Rab11^DN^ construct either in the whole midgut or just ICPs alone caused gaps to appear between the cells (Figs 4g, 5e). However, while blocking endocytosis mimics the effects of E-Cad overexpression, live imaging revealed that blocking recycling on Rab11^DN^ expression affects PMG migration behaviour in a different manner to E-Cad mutants, as both ICP and PMEC migration is stalled by Rab11^DN^ expression (Fig. 4h,i; Supplementary Fig. 5c,d; Supplementary Movie 9).

**Figure 6:**
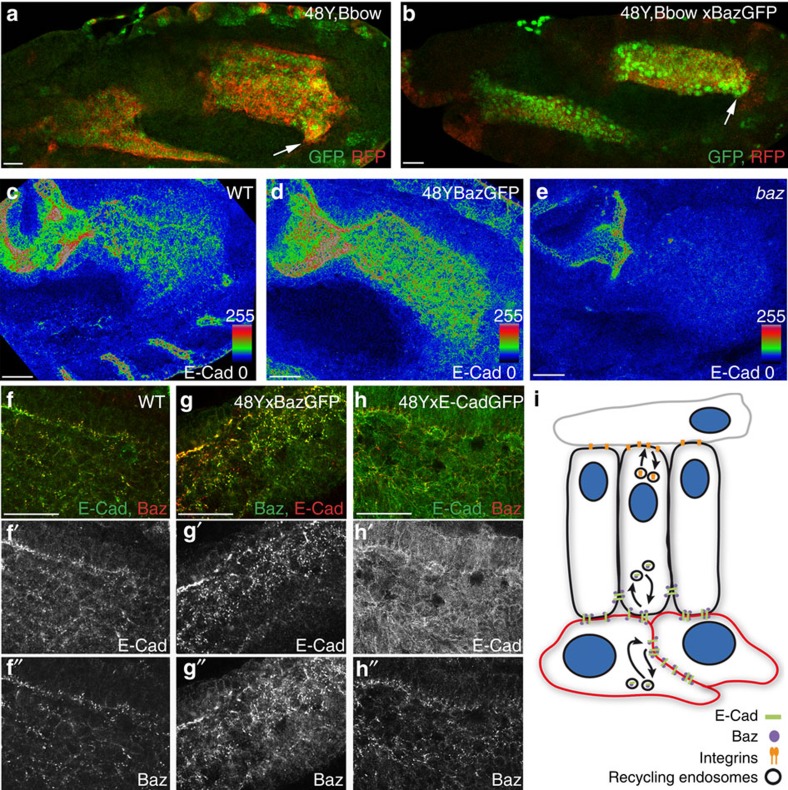
Role of Baz in E-Cad membrane localization. (**a,b**) Mid-stage 12 embryos with 48Y-Gal4 driving expression of either Bbow alone (**a**) or together with Baz-GFP (**b**). Arrows point to the migration front of PMG cells. (**c**–**e**) E-Cad levels are higher throughout the PMG on Baz overexpression (**d**) and markedly decreased in *baz* mutants, which also shows a strong delay in migration. (**e**–**h**) Ectopic Baz-GFP recruits endogenous E-Cad which co-localize in punctae ((**g**) compare with wild type (WT) (**f**)), in contrast endogenous Baz does not co-localize with overexpressed E-Cad-GFP (**h**). (**i**) Illustration of the regulation of E-Cad levels at the membrane through interactions with Baz and the endocytic pathway in both PMECs (black) and ICPs (red); and of the trafficking of integrins mediating dynamic interactions between the PMECs and the mesoderm cells (grey). Scale bar, 20 μm.

From these phenotypes, it is clear that in addition for regulating E-Cad levels, another protein or proteins required for the active migration of both cell types are likely to be trafficked through recycling endosomes. It has previously been shown that the coordination of PMEC behaviour with the mesoderm is mediated by integrins^5^,^6^, and that loss of the βPS integrin subunit, normally expressed in both PMG and visceral mesoderm cells, causes a delay in PMG migration, similar to that seen in Shi^DN^ and Rab11^DN^ conditions. Staining for βPS revealed that in wild-type embryos high punctate accumulations of βPS can be found at the PMG–visceral mesoderm interface, and these are also found in *shg* mutant embryos (Supplementary Fig. 6a–c). However, in both Shi^DN^- and Rab11^DN^-expressing PMGs, this high concentration at the border is lost and βPS is found more uniform throughout the PMECs. Taken together, these data support a model whereby E-Cad and other proteins required for PMG migration, such as the βPS integrin subunit, are constantly endocytosed and recycled in PMG cells, thereby maintaining a balance of transient adhesive interactions both between the midgut cells, and between PMECs and the mesoderm, that act to couple migration of these different cell types, without impeding it (Fig. 6i).

### Baz is required to recruit E-Cad to the membrane of ICPs

In the *Drosophila* embryonic epithelium, one of the earliest cues for recruitment of E-Cad to the apical membrane is the Par3 homologue Bazooka (Baz)^22^, which is itself apicolaterally localized through interactions with other epithelial polarity cues^23^,^24^. It was thus intriguing to note that Baz is also expressed in non-epithelial cells of the midgut throughout migration and shows a strong overlap with E-Cad (Fig. 6f; Supplementary Fig. 1). Furthermore, we found that overexpression of Baz-green fluorescent protein (GFP) in the midgut causes a significant increase in the levels of E-Cad in both PMECs and ICPs, which co-localizes precisely with exogenous Baz-GFP (Fig. 6d,g) and that levels of midgut E-Cad are greatly reduced in *baz* mutant embryos (Fig. 6e). Consistently, overexpression of Baz-GFP has a similar effect on migration behaviour as ectopic E-Cad, as shown by a decrease in migration speeds of both cell types as measured *in vivo* (Fig. 6b; Supplementary Fig. 7; Supplementary Movie 10) and causes a specific delay in ICPs migration when triggered only in these cells (Fig. 5d). In contrast, when E-Cad is overexpressed, there is no effect on the levels of Baz (Fig. 6h), suggesting that while Baz is both necessary and sufficient to recruit E-Cad to the cell membrane, E-Cad does not affect Baz levels.

Interestingly, despite an almost complete loss of E-Cad in the PMGs of *baz* mutants (Fig. 6e), migration of the PMG is severely delayed, it fails to elongate along the visceral mesoderm, and remains very rounded throughout germband retraction (Fig. 6e; Supplementary Fig. 6f). Given the similarity of this phenotype to that of rab11 dominant negative, we decided to investigate the expression of βPS in *baz* mutant PMGs. We found that βPS no longer localizes to the PMG–visceral mesoderm interface, but is found all around the cell surface (Supplementary Fig. 6f). Thus, while it is clear that correct levels of Baz are required in PMG cells to maintain precise levels of membrane E-Cad, it also plays an additional role in localizing βPS to the PMEC–visceral mesoderm border, which is in turn required for the normal migration of the PMECs.

Finally, due to the co-localization of Baz and E-Cad in PMG cells, we next investigated whether Baz is also trafficked through the endocytic pathway. We found that similar to E-Cad, Baz levels are upregulated when endocytosis is blocked in the PMG using ShiDN (Supplementary Fig. 6h), and, conversely, Baz levels are decreased in Rab11DN conditions (Supplementary Fig. 6I). These data further demonstrate the key role of endocytic trafficking in PMG migration, regulating the levels/localization not only of E-Cad and the βPS integrin subunit but also of Baz (Fig. 6i).

## Discussion

Previous studies on the role of E-Cad have focused on homogeneous populations of epithelial cells. E-Cad has long been considered to play a role in their static behaviour, mediating homophilic attachments through adherens junctions and its repression a necessary or even sufficient step for epithelial cells to become migratory in the EMT^8^,^25^. While mutants for E-Cad had been shown to affect some cell migration events^26^,^27^, it has only been very recently that the analysis of cell-specific requirements has shown E-Cad to play an active role in the migration of a certain type of epithelial cells, the *Drosophila* border cells, mediating adhesion, polarizing the cluster and orchestrating the directional migration of the group^28^. Our results indicate a further role for E-Cad as an adhesive factor in the migration of non-epithelial cells based on the following observations. First, in embryos mutant for E-Cad, there is a clear effect on the mesenchymal ICPs, without any obvious defect in the PMECs. Second, in these same embryos, the defects in ICPs are associated with the cell membranes between the ICPs no longer being closely aligned, with large gaps appearing between them. And third, the organization of non-epithelial cells during migration is perturbed when E-Cad function is disrupted just in these cells. Indeed, this could be quite a general role for E-Cad in heterogeneous populations of non-epithelial cells. In fact, the mesendoderm cells of zebrafish have been shown to actually upregulate E-Cad before migration and when E-Cad function in the whole embryo is compromised, they fail to migrate efficiently along the epiblast^27^. Our model of E-Cad recycling could account for this phenotype (Fig. 6h).

Our work also shows that the same mechanisms that act on the epithelial control of E-Cad membrane accumulation are also active in non-epithelial cells. This notion is strengthened by our results showing that a ‘polarity protein' such as Baz/Par3 is instructive in the recruitment of E-Cad in non-apicobasally polarized cells. Together, these results indicate that the adhesive role of E-Cad and the mechanisms regulating its recycling to the membrane could be shared by both epithelial and non-epithelial cells whether or not E-Cad is organized into junctional structures. Indeed, the adhesive function of E-Cad in these endodermal cells is not localized to a specific domain, as our ultrastructural analysis shows that in the absence of E-Cad, the tight membrane alignment of ICP cells is perturbed all around the cell circumference. We suggest that the epithelial specificity for junctions could instead be more related to ‘extra-adhesive functions' of E-Cad such as cell polarization and intercellular cytoskeleton coupling as suggested in the review^12^. Finally, we would like to note that the morphogenesis of many endodermal organs, such as the thyroid, liver and pancreas, critically depends on interactions between endodermal and mesenchymal cells types^29^,^30^. Furthermore, almost all human tumours comprise heterogenous populations of epithelial and non-epithelial cells. Thus, our study suggests a potential role for E-Cad in coupling the behaviour of both cell types in many different developmental and pathogenic contexts.

## Methods

### Fly strains and genetics

Details for all genotypes and transgenes can be found in flybase (http://flybase.org) or in references listed here. Unless otherwise noted, stocks were obtained from the Bloomington Stock centre. Wild-type embryos were from *yw* stocks. The Gal4/UAS system^31^ was used to drive the expression of transgenes. Transgenes were driven in the PMG using either the hkbGal4 or the 48Y-Gal4 drivers recombined with UAS-GMA-GFP, UAS-Stinger or UAS-Brainbow. To misexpress in the ICPs, an insc-Gal4 recombined with UAS-SrcGFP was used. Within the midgut, the insc-Gal4 driver is specific for the ICPs and AMPs, but it also drives expression in other tissues such as the nervous system. The mutant strain for E-Cad used was the strong *shg* allele, *shg*^*G317*^ (ref. 10), and PMG cells were visualized by recombining this allele with 48Y,stingerGFP, 48Y,GMAGFP or 48Y,Brainbow. To interfere with E-Cad function specifically in ICP cells, UAS-DE-cad^ex^ was used^14^. To overexpress E-Cad, UAS-dE-CadGFP was used. To interfere with endocytic trafficking, we used the strong UAS-Shi44A(3-10) transgene^32^; and Rab11DN (provided by Marcos González-Gaitán). To study the requirement for Baz, the allele *baz*^xi106^ and transgene UAS-Baz-GFP were used (provided by Daniel St Johnston). For all ectopic expression experiments, we maximized expression by collecting embryos at 29 °C.

### Immunohistochemistry, fixed image acquisition and analysis

Embryos were fixed, mounted and staged using standard techniques. Antibodies used were as follows: rabbit anti-Baz (1:1,000; gift from A. Wodarz); mouse anti-βPS integrin (CF.6G11; 1:20; Hybridoma Bank); rat anti-E-Cad (DCAD2; 1:100; Hybridoma Bank); mouse anti-FasIII (7G10; 1:20; Hybridoma Bank); guinea pig anti-Fkh (1:250; H, Jaeckle); goat anti-GFP (AB6673; 1:500; Abcam); mouse anti-Hnt (1G9; 1:20; Hybridoma Bank); rabbit anti-Insc (1:500; gift from W. Chia); rabbit anti-RFP (A11122; 1:300; Life Technologies). For labelling with anti-E-Cad, embryos were fixed in 4% paraformaldehyde for just 10 min. For all other stainings, embryos were fixed using standard techniques. Cy2-, Cy3- and Cy5-conjugated secondary antibodies were from Molecular Probes and were used at 1:200 dilutions. Confocal images were acquired using a Leica SP5.

### Electron microscopy

Embryos were staged to 7.5–8 h, dechorionated, fixed with 25% glutaraldehyde in heptane, hand peeled in PBS and then prepared for transmission electron microscopy using standard techniques^33^. Ultrathin sections were analysed using a Tecnai SPIRIT transmission electron microscope.

### Live imaging

Embryos were dechorinated using bleach and stage 10 embryos were manually picked from an agar plate using a Leica fluorescent dissecting microscope. The selected embryos were dorsally or laterally orientated and mounted on a coverslip coated with heptane glue to prevent drift during imaging. A drop of oil was placed on the embryos to maintain their survival. Embryos were imaged using an inverted Leica SP5 2-Photon microscope at 890-nm wavelength, using a 60 × oil immersion lens. Multi-position time-lapse stacks of 20–25 μm, and z-depth of 1.5 was acquired at 2-min intervals over a period of 60 min. Six movies per condition were selected for analysis, and the starting point was defined as the initiation of germband retraction, which is unaffected in the different conditions.

### Time-lapse preprocessing

The cells under study can exhibit a fast directed movement and are densely packed, making their tracking challenging. The overall movement can be broadly estimated and advantageously compensated for to lower the burden on the tracker. This was performed using a custom ImageJ macro that incrementally and uniformly shifts all the images from each frame to partially cancel out the estimated overall displacement. The shifts applied to the images are stored and accounted for at a later stage.

### Nuclei tracking

The nuclei tracking was performed from the fluorescent signal of the nuclei using FIJI plugin Trackmate. This plugin implements a blob detector based on an adjusted 3D Laplacian of Gaussian filter followed by 3D local minima detection. The candidate nuclei are selected based on the intensity at the local minima and the distance to the closest local minima: if this distance is closer to the characteristic radius of the nuclei, only the strongest minima is kept. This last criterion vastly improved the results of the tracking and was implemented as a custom extension to Trackmate. To link the nuclei and build their tracks, the plugin then relies on a frame to frame constrained linear assignment.

### Tracks visualization and correction

The nuclei tracks were exported from Trackmate to spreadsheets and the original overall movement was numerically restored by accordingly shifting the positions of the detected blobs. Each track was then manually checked. To this end, we developed a custom ImageJ macro allowing the overlay of tracks originating from a specific region of interest to the original movie. With this tool, we were able to follow a nucleus along a track in 3D, as the z-slice is automatically adjusted to the detected position. Tracks from ICPs and PMECs were selected according to the nuclear diameter: PMECs <3.5 μm and ICPs >5.5 μm. Only the valid tracks starting inside the specified regions and spanning the whole movie were kept, yielding an average of 15 tracks of each cell type per movie, corresponding to some 300 nuclei positions defined over time per embryo.

### Tracks statistics

From the selected tracks, the cells instantaneous speeds were estimated from the nuclei frame to frame displacements. A metric of cohesion of the local movements was estimated as the correlation of a nucleus instantaneous speed direction (and magnitude) and that of the nucleus from the closest track (track distance evaluated at the first time point; Fig. 2e,f). From this information, the track average velocity, coordination and directional persistence were derived. Finally, the average and s.d. of these measurements were computed over all the tracks of all the movies from the same condition.

## Additional information

**How to cite this article:** Campbell, K. *et al.* A role for E-cadherin in ensuring cohesive migration of a heterogeneous population of non-epithelial cells. *Nat. Commun.* 6:7998 doi: 10.1038/ncomms8998 (2015).

## Supplementary Material

Supplementary Information Supplementary Figures 1-7 and Supplementary Table 1

Supplementary Movie 1 Time-lapse movie of an embryo expressing UAS-SrcGFP under the control of the ICP specific driver, InscGal4. The movie begins mid germband retraction, frames are taken every 2 mins and a maximum projection of 2 z-slices of 1.5μm thickness is shown.

Supplementary Movie 2 Time-lapse movie of the wild type behaviour of migrating PMG cells. The movie begins at the initiation of germband retraction and actin is visualised by UAS-GMA-GFP, whose expression is driven by the general PMG driver 48Y-Gal4. A maximum projection of 3 z-slices of 1.5μm thickness is shown. Frames are taken every 2 mins.

Supplementary Movie 3 Time-lapse movie of migrating PMG cells in a shgG315 embryo. The movie begins at the initiation of germband retraction and actin is visualised by UAS-GMA-GFP, whose expression is driven by the general PMG driver 48Y-Gal4. A maximum projection of 3 z-slices of 1.5μm thickness is shown. Frames are taken every 2 mins.

Supplementary Movie 4 An example of a time-lapse movie that was used for the tracking of migrating PMG cells in wild type embryos. PMG cell nuclei were labelled by StingerGFP. The movie begins at the initiation of germband retraction, frames are taken every 2 mins, a maximum projection of 2 z-slices of 1.5μm thickness is shown.

Supplementary Movie 5 An example of a time-lapse movie that was used for the tracking of migrating PMG cells in shgG315 mutant embryos. PMG cell nuclei were labelled by StingerGFP. The movie begins at the initiation of germband retraction, frames are taken every 2 mins, a projection of 2 z-slices of 1.5μm thickness is shown.

Supplementary Movie 6 An example of a time-lapse movie that was used for the tracking of migrating PMG cells in embryos expressing DE-cad.ex in all midgut cells. PMG cell nuclei were labelled by StingerGFP. The movie begins at the initiation of germband retraction, frames are taken every 2 mins, a projection of 2 z-slices of 1.5μm thickness is shown.

Supplementary Movie 7 An example of a time-lapse movie that was used for the tracking of migrating PMG cells in embryos overexpressing E-CadGFP in all midgut cells. PMG cell nuclei were labelled by StingerGFP. The movie begins at the initiation of germband retraction, frames are taken every 2 mins, a projection of 2 z-slices of 1.5μm thickness is shown.

Supplementary Movie 8 An example of a time-lapse movie that was used for the tracking of migrating PMG cells in embryos expressing ShiDN in all midgut cells. PMG cell nuclei were labelled by StingerGFP. The movie begins at the initiation of germband retraction, frames are taken every 2 mins, a projection of 2 z-slices of 1.5μm thickness is shown.

Supplementary Movie 9 An example of a time-lapse movie that was used for the tracking of migrating PMG cells in embryos expressing Rab11DN in all midgut cells. PMG cell nuclei were labelled by StingerGFP. The movie begins at the initiation of germband retraction, frames are taken every 2 mins, a projection of 2 z-slices of 1.5μm thickness is shown.

Supplementary Movie 10 An example of a time-lapse movie that was used for the tracking of migrating PMG cells in embryos expressing BazGFP in all midgut cells. PMG cell nuclei were labelled by StingerGFP. The movie begins at the initiation of germband retraction, frames are taken every 2 mins, a projection of 2 z-slices of 1.5μm thickness is shown.
