# Supplementary material for: A role for E-cadherin in ensuring cohesive migration of a heterogeneous population of non-epithelial cells
Source: Nat Commun. 2015 Aug 14;6:7998. doi: 10.1038/ncomms8998 (PMC4557277; doi:10.1038/ncomms8998)
Supplement: Supplementary Information — Supplementary Figures 1-7 and Supplementary Table 1 [file ncomms8998-s1.pdf]

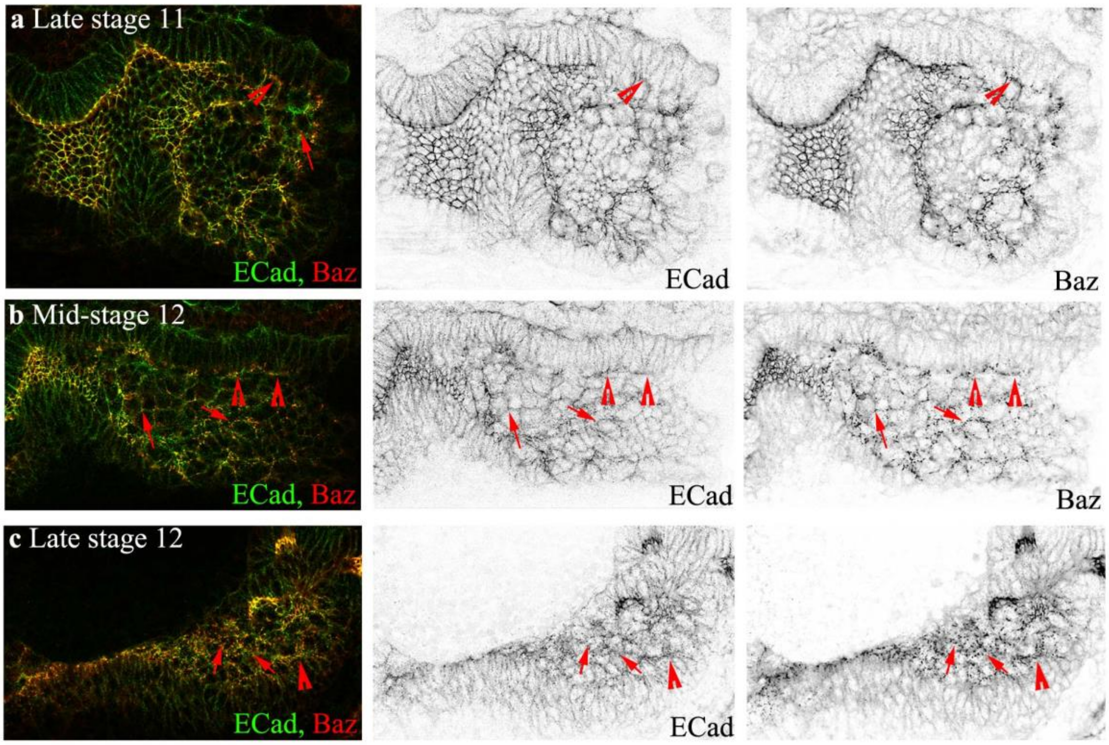

Supplementary Figure 1. E-Cad and Baz are expressed in both ICPs and PMECs throughout midgut migration. **a,b,c,** WT embryos stained for E-Cad (green) and Baz (red) at late stage 11 **(a)**, mid-stage 12 **(b)** and late stage 12 **(c).** Arrowheads point to PMECs and arrows to ICPs.

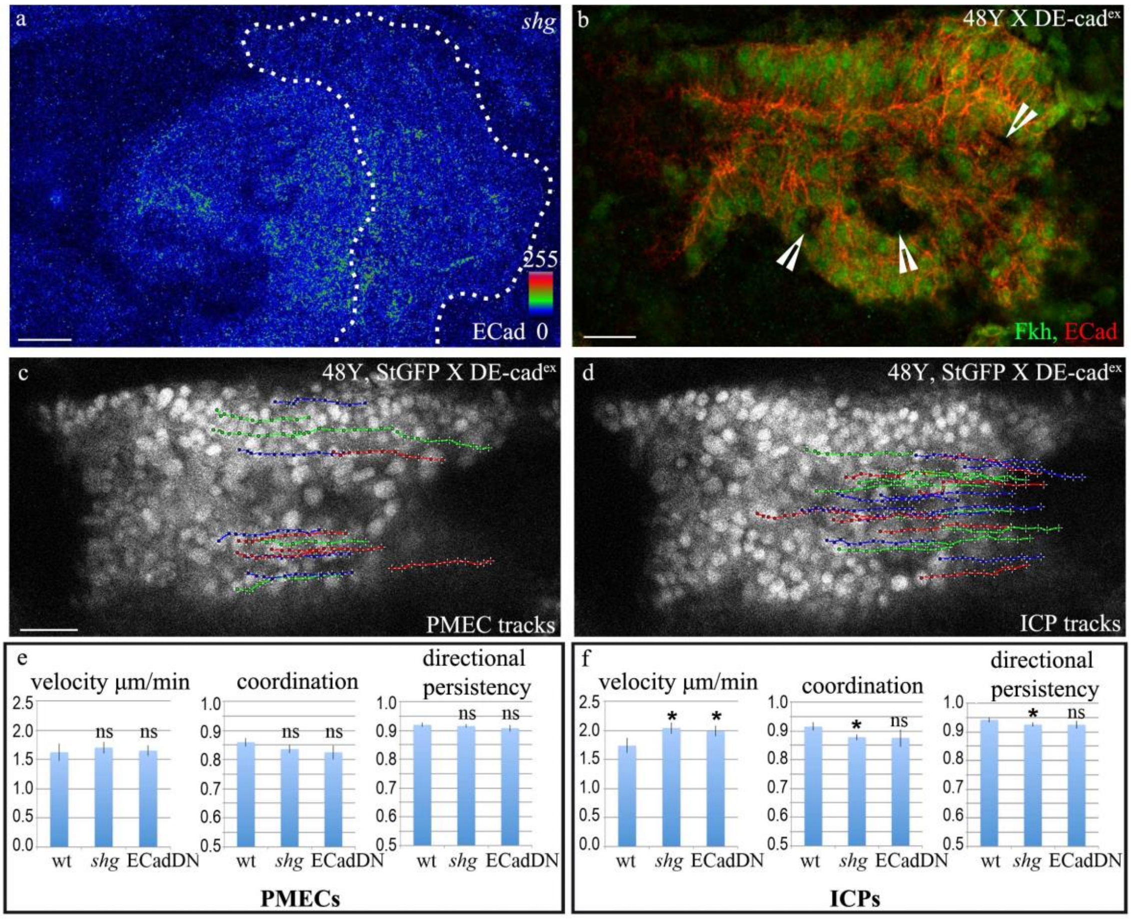

Supplementary Figure 2. Expression of UAS-DE-cad.ex in the PMG produces phenotypes similar to *shg* mutants. **(a)** ECad expression is reduced to very low levels in the migrating PMG of the *shg*^{G317} allele. **(b)** Stage 12 embryo stained for Fkh (green) to visualise the PMG cells, and E-Cad (red), arrowheads point to holes between the cells. **(c,d)** Tracks representative of the paths taken by PMECs **(c)** and ICPs **(d)**. **(e,f)** Velocity, coordination and directional persistence values calculated from movies of DE-cad.ex expressing PMG. Data are presented as mean ± SEM.*$p<0.05$; ns=not significant; by paired t-test, n=6 for each condition (see Supplementary Table 1 for raw data). Scale bars:20µm.

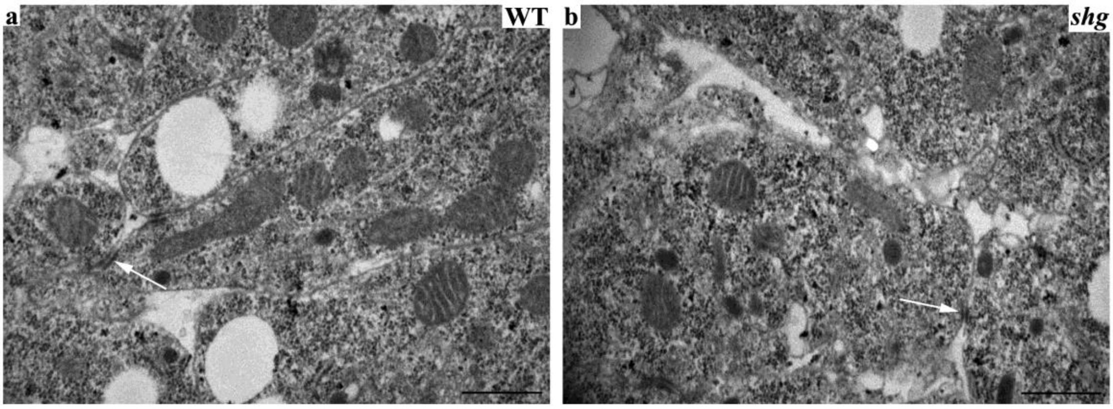

Supplementary Figure 3. Spot adherens junctions are found between a few ICPs in both WT and *shg* mutants. **a,** Transmission electron microscope images of ICPs in WT embryos show that there are only a few scattered spot adherens junctions between a small number of ICPs (arrow); from 136 cells analysed, 8 spot adherens junctions were found. **b,** These can also be found in *shg* mutants (arrow); from 87 cells analysed, 4 spot adherens junctions were found. Scale bars: 500nm.

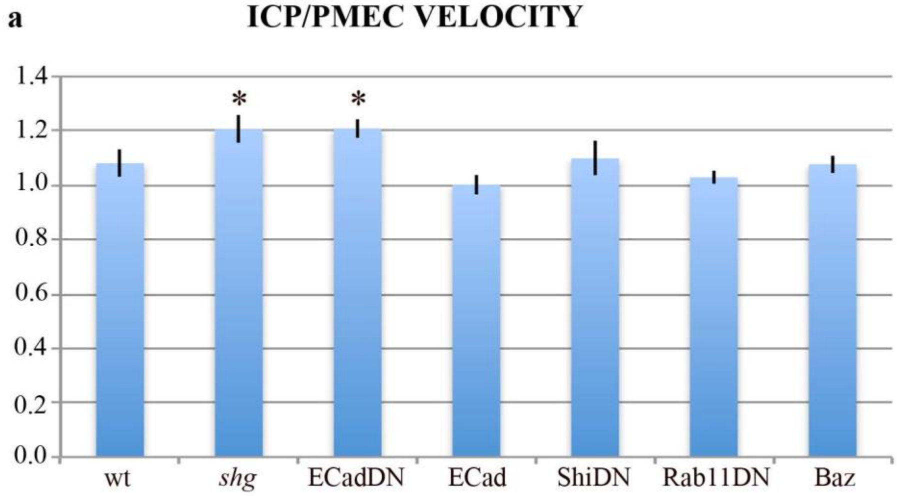

Supplementary Figure 4. Analysis of the ratio of PMEC and ICP velocities. **a.** Data are presented as mean ±SEM. *p<0.05, by paired t-test, n=6 for each condition (see Supplementary Table 1 for raw data).

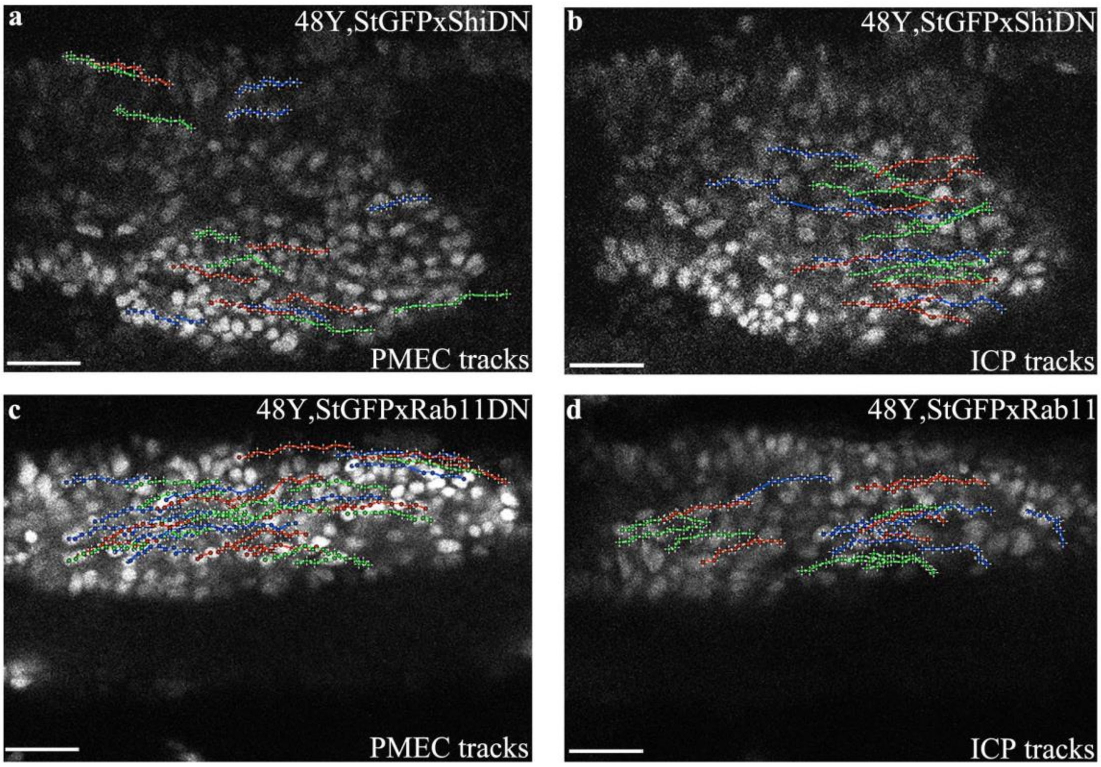

Supplementary Figure 5. Representative tracks of the paths taken by PMECs and ICPs when endocytic trafficking is perturbed throughout the midgut. **a-d,** PMECs and ICPs are identified by their nuclear diameter (PMECs <3.5μm, ICPs >5.5μm). In order to aid comparison, tracks are arbitrarily labelled in red, blue and green. **a,b,** PMEC **(a)** and ICP **(b)** tracks when ShiDN is driven together with StGFP by the general midgut driver 48YGal4. **c,d,** PMEC **(c)** and ICP **(d)** tracks when Rab11DN is driven together with StGFP by 48YGal4. Scale bars:20μm.

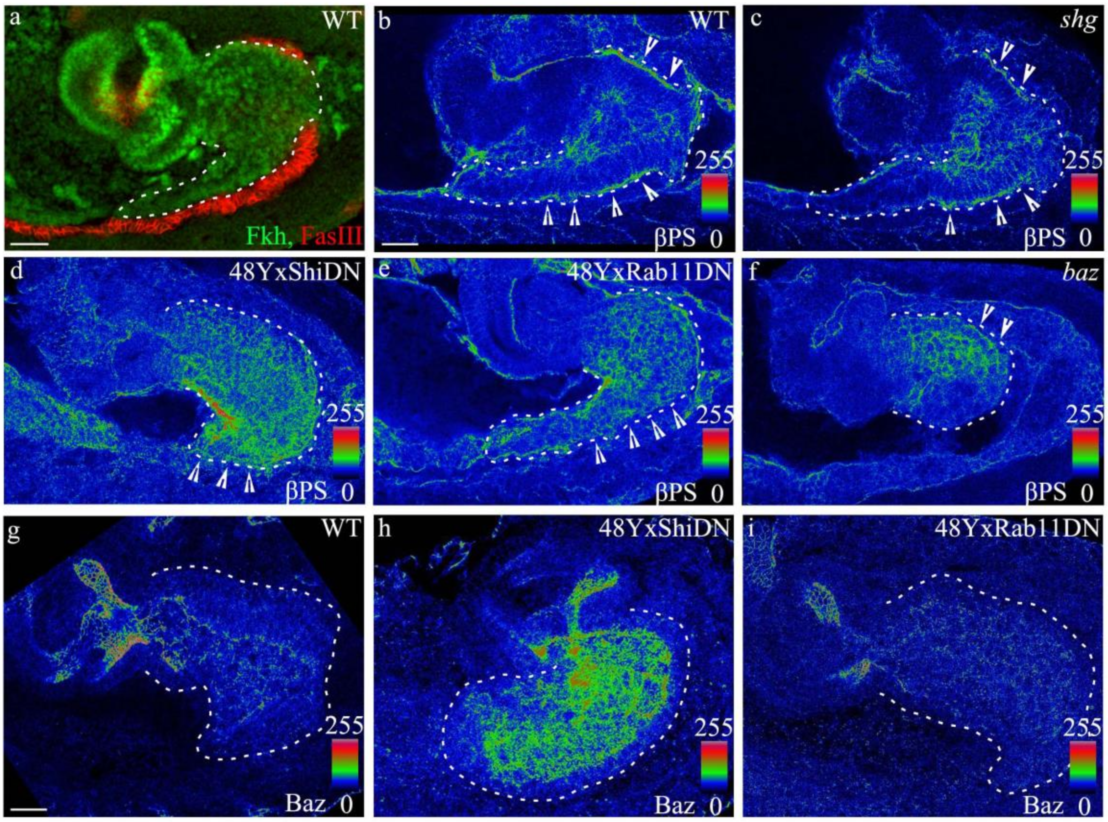

Supplementary Figure 6. Endocytic trafficking is required for the correct localisation of βPS integrin and levels of Baz during PMG migration. **a-i** stage 12 embryos. Genotypes are WT **(a,b,g)**; shg **(c)**; 48Y;UAS-ShiDN **(d,h)**; 48Y;UAS-Rab11DN **(e,i)** and baz **(f)**. **(a)** During migration the PMG (visualised with Fkh (green)) migrates along the visceral mesoderm (visualised with FasIII (red)). **b-i** show a colorimetric readout of βPS **(b-f)** or Baz **(g-i)** levels. **(b,c)** βPS integrin is found highly concentrated in puntae in the PMECs at the PMG/visceral mesoderm boundary (**b**, arrowheads), and this is not perturbed in shg mutants (**c**, arrowheads). **(d,e)** Upon ectopic ShiDN **(d)** or RabllDN **(e)**, higher concentrations of βPS are no longer found at the PMG/visceral mesoderm boundary. **(f)** In *baz* mutant embryos βPS is found delocalised througout the PMECs. **(g-i)** In comparison to WT **(g)**, levels of Baz are increased in the PMG upon ectopic ShiDN **(h)** and decreased upon ectopic RabllDN **(i)**. Scale bars: 20µm.

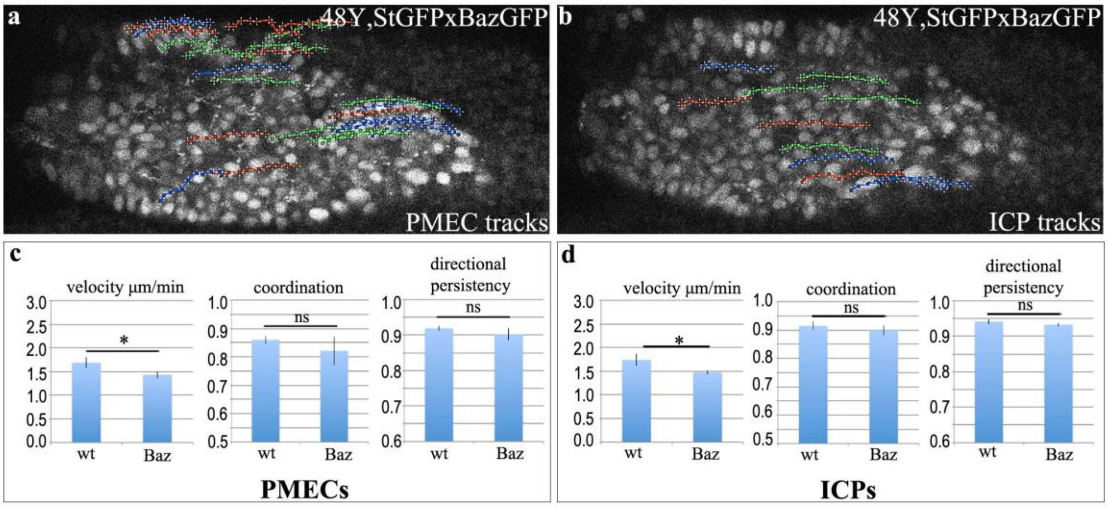

Supplementary Figure 7. Overexpression of Baz causes a delay in the migration of both PMECs and ICPs. **a,b,** Tracks representative of the paths taken by PMECs **(a)** and ICPs **(b)** with increased levels of Baz. **c,d,** Velocity, coordination and directional persistence values calculated from movies made of WT and Baz overexpressing PMGs. Data are presented as mean ±SEM. *$p<0.05$; ns= not significant; by paired t-test, n=6 for each condition (see Supplementary Table 1 for raw data).

WT	ICP velocity μm/min	PMEC velocity μm/min	ICP/PMEC ratio	ICP dir persistency	PMEC dir persistency	ICP coordination	PMEC coordination
mov 1	1.809	1.623	1.114	0.950	0.928	0.927	0.880
mov 2	1.629	1.623	1.004	0.916	0.918	0.877	0.891
mov 3	1.581	1.498	1.055	0.948	0.897	0.928	0.864
mov 4	1.574	1.603	0.982	0.936	0.917	0.885	0.807
mov 5	2.223	2.135	1.041	0.961	0.939	0.950	0.868
mov 6	1.603	1.644	0.975	0.938	0.914	0.922	0.847
average	1.736	1.688	1.029	0.941	0.919	0.915	0.860
s.d.	0.254	0.225	0.053	0.015	0.014	0.028	0.030
shg							
mov 1	2.231	1.907	1.170	0.937	0.921	0.877	0.872
mov 2	1.974	1.411	1.399	0.929	0.918	0.880	0.808
mov 3	2.062	1.738	1.186	0.933	0.893	0.892	0.800
mov 4	1.799	1.596	1.127	0.917	0.919	0.886	0.853
mov 5	1.870	1.627	1.150	0.900	0.921	0.838	0.828
mov 6	2.304	1.919	1.201	0.935	0.919	0.894	0.854
average	2.040	1.700	1.206	0.925	0.915	0.878	0.836
s.d.	0.199	0.196	0.098	0.014	0.011	0.020	0.029
p-value	**0.022**	0.298	**0.033**	**0.043**	0.302	**0.013**	0.092
**DE-cad.ex**							
mov 1	1.779	1.427	1.247	0.940	0.912	0.877	0.830
mov 2	2.132	1.891	1.127	0.949	0.936	0.930	0.890
mov 3	2.195	1.716	1.279	0.884	0.916	0.818	0.826
mov 4	1.976	1.665	1.187	0.946	0.907	0.934	0.835
mov 5	1.769	1.443	1.226	0.025	0.911	0.905	0.829
mov 6	2.075	1.751	1.185	0.902	0.862	0.787	0.739
average	1.987	1.649	1.208	0.924	0.907	0.875	0.825
s.d.	0.180	0.182	0.054	0.026	0.024	0.061	0.048
p-value	**0.038**	0.434	**0.015**	0.100	0.169	0.090	0.080
**E-CadGFP**							
mov 1	1.559	1.487	1.049	0.862	0.859	0.806	0.773
mov 2	1.386	1.463	0.947	0.833	0.685	0.715	0.473
mov 3	1.303	1.264	1.031	0.926	0.906	0.836	0.794
mov 4	1.299	1.244	1.044	0.751	0.751	0.605	0.652
mov 5	1.308	1.232	1.062	0.887	0.797	0.727	0.666
mov 6	1.320	1.499	0.881	0.760	0.833	0.408	0.740
average	1.363	1.365	1.002	0.837	0.805	0.683	0.683
s.d.	0.102	0.130	0.072	0.070	0.079	0.157	0.118
p-value	**0.004**	**0.006**	0.245	**0.002**	**0.003**	**0.003**	**0.003**
**ShiDN**							
mov 1	1.385	1.397	0.991	0.926	0.894	0.845	0.867
mov 2	1.056	1.041	1.015	0.879	0.874	0.830	0.813
mov 3	1.540	1.209	1.274	0.913	0.861	0.836	0.698
mov 4	1.569	1.408	1.114	0.908	0.871	0.851	0.833
mov 5	1.311	1.332	0.984	0.836	0.831	0.784	0.800
mov 6	1.347	1.404	0.959	0.922	0.896	0.865	0.840
average	1.368	1.299	1.056	0.897	0.871	0.835	0.808
s.d.	0.185	0.147	0.120	0.034	0.024	0.028	0.059
p-value	**0.009**	**0.003**	0.309	**0.012**	**0.001**	**0.000**	**0.048**
**Rab11DN**							
mov 1	1.428	1.437	0.993	0.968	0.939	0.920	0.896
mov 2	1.270	1.334	0.952	0.878	0.914	0.836	0.929
mov 3	1.415	1.293	1.095	0.951	0.925	0.928	0.869
mov 4	1.569	1.509	1.040	0.953	0.951	0.906	0.916
mov 5	1.084	1.039	1.043	0.953	0.926	0.861	0.878
mov 6	1.102	1.050	1.050	0.915	0.930	0.802	0.891
average	1.311	1.277	1.029	0.936	0.931	0.875	0.897
s.d.	0.194	0.195	0.050	0.033	0.013	0.051	0.023
p-value	**0.004**	**0.004**	0.496	0.368	0.084	0.063	**0.018**
**BazGFP**							
mov 1	1.436	1.268	1.132	0.917	0.907	0.862	0.849
mov 2	1.508	1.557	0.969	0.922	0.907	0.887	0.886
mov 3	1.502	1.400	1.073	0.935	0.960	0.913	0.939
mov 4	1.437	1.308	1.098	0.939	0.865	0.870	0.689
mov 5	1.610	1.445	1.114	0.943	0.903	0.960	0.852
mov 6	1.374	1.616	0.850	0.939	0.868	0.899	0.712
average	1.478	1.433	1.039	0.933	0.902	0.898	0.821
s.d.	0.082	0.136	0.109	0.010	0.035	0.036	0.099
p-value	**0.027**	**0.022**	0.417	0.138	0.148	0.203	0.198

SUPPLEMENTARY TABLE 1. Analysis of velocity, ICP/PMEC ratio, directional persistency and coordination. The data are presented as the mean values of an average of 15 tracks per cell type, per movie. P-values were calculated using a paired student's t-test comparing the WT and the mutant condition under consideration.
